# Investigation of Receptor Heteromers Using NanoBRET Ligand Binding

**DOI:** 10.3390/ijms22031082

**Published:** 2021-01-22

**Authors:** Elizabeth K. M. Johnstone, Heng B. See, Rekhati S. Abhayawardana, Angela Song, K. Johan Rosengren, Stephen J. Hill, Kevin D. G. Pfleger

**Affiliations:** 1 Molecular Endocrinology and Pharmacology Group, Harry Perkins Institute of Medical Research, Nedlands, WA 6009, Australia; ethan.see@perkins.uwa.edu.au (H.B.S.); rekhati.abhaya@perkins.uwa.edu.au (R.S.A.); Steve.Hill@nottingham.ac.uk (S.J.H.); 2 Centre for Medical Research, The University of Western Australia, Crawley, WA 6009, Australia; 3 Australian Research Council Centre for Personalised Therapeutics Technologies, Canberra, NSW 2609, Australia; 4 School of Biomedical Sciences, Faculty of Medicine, The University of Queensland, St Lucia, QLD 4072, Australia; a.song@uq.edu.au (A.S.); j.rosengren@uq.edu.au (K.J.R.); 5 Division of Physiology, Pharmacology and Neuroscience, School of Life Sciences, University of Nottingham Medical School, Nottingham NG7 2UH, UK; 6 Centre of Membrane Proteins and Receptors, University of Nottingham, Midlands NG7 2UH, UK; 7 Dimerix Limited, Nedlands, WA 6009, Australia

**Keywords:** NanoBRET, Nluc, GPCR, heteromer, ligand binding, Receptor-HIT, angiotensin receptor, β_2_ adrenergic receptor

## Abstract

Receptor heteromerization is the formation of a complex involving at least two different receptors with pharmacology that is distinct from that exhibited by its constituent receptor units. Detection of these complexes and monitoring their pharmacology is crucial for understanding how receptors function. The Receptor-Heteromer Investigation Technology (Receptor-HIT) utilizes ligand-dependent modulation of interactions between receptors and specific biomolecules for the detection and profiling of heteromer complexes. Previously, the interacting biomolecules used in Receptor-HIT assays have been intracellular proteins, however in this study we have for the first time used bioluminescence resonance energy transfer (BRET) with fluorescently-labeled ligands to investigate heteromerization of receptors on the cell surface. Using the Receptor-HIT ligand binding assay with NanoBRET, we have successfully investigated heteromers between the angiotensin II type 1 (AT_1_) receptor and the β_2_ adrenergic receptor (AT_1_-β_2_AR heteromer), as well as between the AT_1_ and angiotensin II type 2 receptor (AT_1_-AT_2_ heteromer).

## 1. Introduction

Receptor heteromerization is the formation of a complex composed of two (or more) functional receptor units. Although the constituent receptors are functional in their own right as monomers/homomers, the formation of a heteromer results in a complex that may have distinct biochemical properties [1]. This attainment of novel heteromer pharmacology expands the complexity of receptor signaling networks and adds selectivity and specificity to receptor signaling. Although this makes receptor heteromers exciting potential drug targets, to date only modest progress has been made towards therapeutically targeting these complexes. One reason for this is the difficulty in detecting heteromers and monitoring their pharmacology. In particular, differentiating heteromer-specific pharmacology from monomer/homomer-specific pharmacology can be a major challenge. In order to address this issue, we have developed the Receptor-Heteromer Investigation Technology (Receptor-HIT) [2,3], which has been used to investigate several G protein-coupled receptor (GPCR) heteromers [2,4,5,6,7,8,9,10,11]. In addition, the Receptor-HIT assay has been used to characterize receptor tyrosine kinase (RTK) heteromers [12], as well as heteromers composed of both GPCRs and RTKs [13,14,15] or a GPCR and the Receptor for Advanced Glycation End-products [16].

**Figure 1:**
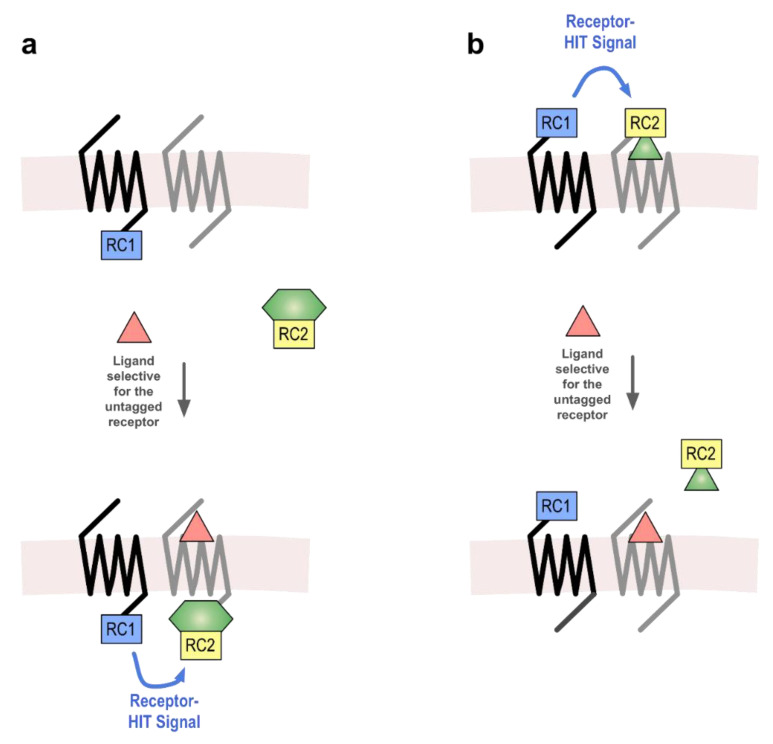
Intracellular and extracellular Receptor-Heteromer Investigation Technology (Receptor-HIT) assay**.** Receptor-HIT allows for investigation of receptor heteromers through ligand-dependent modulation of interactions between receptors and specific biomolecules. The Receptor-HIT assay uses a proximity-based reporter system such as bioluminescence or fluorescence resonance energy transfer (BRET or FRET) or protein complementation. The assay has most commonly been used to investigate GPCR heteromers (GPCR-HIT) [2] however it can be used to investigate any type of heteromers, such as RTK heteromers (RTK-HIT) [12]. The assay works by coexpressing each receptor in cells, one receptor tagged with the first reporter component of the proximity assay (RC1), such as a BRET donor, and the other receptor untagged with respect to the proximity assay system. Instead, an interacting biomolecule is tagged with the second reporter component (RC2), such as a BRET acceptor. Usually, this interacting biomolecule is a coexpressed intracellular protein (**a**) however it can also be a labeled ligand (**b**). The assay works by adding a ligand that is selective for the untagged receptor. If this results in modulation of the proximity signal between the tag on the receptor and the tag on the interacting biomolecule, this indicates heteromerization of the two receptors.

The Receptor-HIT assay enables rapid identification, screening and profiling of receptor heteromers through ligand-dependent modulation of interactions between the receptors and specific biomolecules. The assay uses a proximity-based reporter system, such as bioluminescence resonance energy transfer (BRET), which allows the proximity between the receptors and the interacting biomolecules to be monitored. Previously, the interacting biomolecules used in Receptor-HIT studies have been intracellular proteins (Figure 1a), enabling functional interactions to be identified. However, as we have previously discussed [17], Receptor-HIT can also be configured to investigate heteromers at their extracellular surface, with the use of a fluorescent ligand as the labeled interacting biomolecule (Figure 1b).

Recently, we have developed the NanoBRET ligand binding assay [18,19,20,21,22,23], in which we have successfully monitored binding of fluorescent ligands to Nanoluciferase (Nluc)-tagged GPCRs. In the present study, we have demonstrated the Receptor-HIT assay utilizing NanoBRET ligand binding to investigate two GPCR heteromers: between the angiotensin II type 1 (AT_1_) receptor and the β_2_ adrenergic receptor (AT_1_-β_2_AR heteromer), as well as between the AT_1_ and angiotensin II type 2 receptor (AT_1_-AT_2_ heteromer). These three receptors and the hormones that target them have functions in many different physiological systems, and in particular, they have fundamental roles in the maintenance of cardiovascular homeostasis. AT_1_-β_2_AR heteromers have been described in several studies [9,24,25] and are involved in the mediation of cardiomyocyte contractility and heart rate in intact mice [24]. The pharmacology of the AT_1_-AT_2_ heteromer has been extensively studied [4,26,27,28,29,30,31,32,33,34,35], and most recently, has been implicated in Parkinson’s disease [27].

## 2. Results

### 2.1. AT_1_-β_2_AR Heteromer

**Figure 2:**
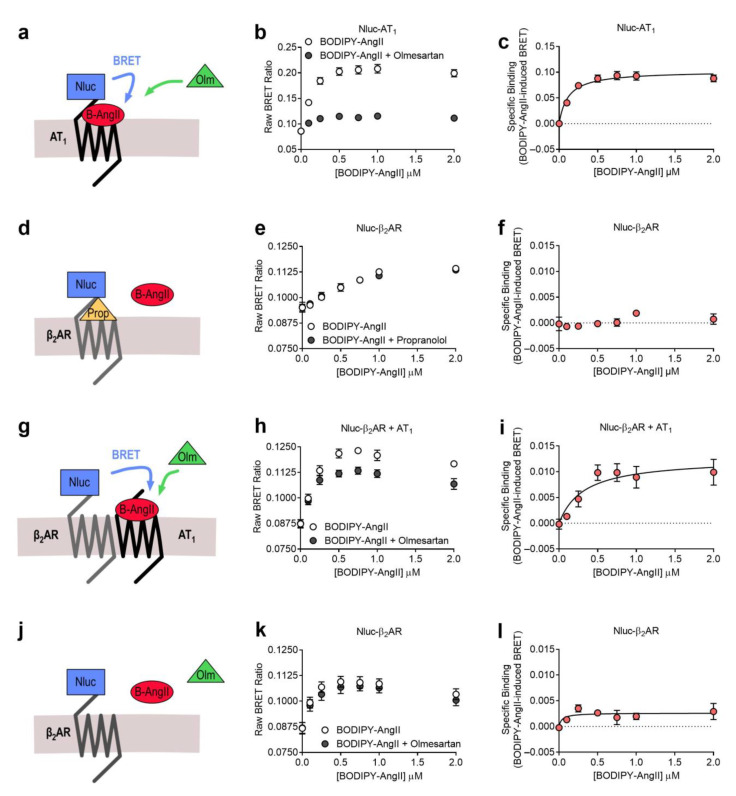
Saturation binding of BODIPY-AngII to the AT_1_ receptor and the AT_1_-β_2_AR heteromer. Cells expressing Nluc-AT_1_ (**a**–**c**) or Nluc-β_2_AR (**d**–**f**,**j**–**l**) or Nluc-β_2_AR + AT_1_ (**g**–**i**) were treated with BODIPY-AngII (B-AngII) in the presence or absence of 1 μM olmesartan (Olm; **a**–**c**,**g**–**l**) or the presence or absence of 10 μM propranolol (Prop; **d**–**f**) to generate BODIPY-AngII total and non-specific binding data (**b**,**e**,**h**,**k**) and specific binding curves (where possible; **c**,**f**,**i**,**l**). Data are displayed as the raw BRET ratio (**b**,**e**,**h**,**k**) or specific binding (**c**,**f**,**i**,**l**), mean ± SEM of three (**e**,**f**) or four (**b**,**c**,**h**,**i**,**k**,**l**) independent experiments.

The first heteromer that we investigated was between the AT_1_ receptor and the β_2_AR. Several studies have investigated this heteromer in a variety of systems, and reported evidence for it being a constitutive heteromer that displays modulated G protein signaling and β-arrestin recruitment [9,24,25]. To investigate this complex we first looked at binding of a BODIPY-630/650 tagged angiotensin II ligand (BODIPY-AngII) to the N-terminally Nluc-tagged AT_1_ receptor (Nluc-AT_1_; Figure 2a) using the NanoBRET ligand binding assay [18,19,20,21]. We were able to demonstrate saturable binding of BODIPY-AngII to the AT_1_ receptor, which was substantially reduced by treatment with a high concentration of the AT_1_ antagonist olmesartan (Figure 2b), enabling generation of a BODIPY-AngII specific binding curve (Figure 2c). To confirm the specificity of our BODIPY-AngII ligand we next conducted the saturation assay in cells expressing *N*-terminally Nluc-tagged β_2_AR (Nluc-β_2_AR) in the presence and absence of the β_2_AR antagonist propranolol (Figure 2d). Here we saw there was no displacement of BODIPY-AngII binding in the presence of propranolol (Figure 2e) and therefore no specific binding of BODIPY-AngII to Nluc-β_2_AR (Figure 2f). In contrast, when we coexpressed the untagged AT_1_ receptor with Nluc-β_2_AR, using the Receptor-HIT assay configuration (Figure 2g), we observed displacement of BODIPY-AngII binding in the presence of olmesartan (Figure 2h) resulting in clear, saturable, specific binding of BODIPY-AngII (Figure 2i). It is important to note here the difference in scale of the *y*-axis, as the BODIPY-AngII-induced BRET signal is approximately ten times stronger between BODIPY-AngII and AT_1_ receptors than BODIPY-AngII and AT_1_-β_2_AR heteromers. This is most likely due to the increased distance between BODIPY-AngII bound to AT_1_ and Nluc-labeled β_2_AR across the AT_1_-β_2_AR heteromer, compared to BODIPY-AngII bound to Nluc-labeled AT_1_ receptors. This specific and saturable binding of BODIPY-AngII in cells expressing Nluc-β_2_AR + AT_1_ confirms the close proximity of the AT_1_ receptor and β_2_AR in our system. Interestingly, when we compared the affinity of BODIPY-AngII binding in the two systems, we saw a small but significant decrease in the affinity of binding to AT_1_-β_2_AR heteromers when compared to binding to AT_1_ receptors (mean *K*_d_ ± SEM = 338.4 ± 57.4 nM vs. 114.1 ± 9.9 nM, respectively; *n* = 4, *p* < 0.05 for unpaired *t*-test). Consistent with the lack of propranolol displacement by BODIPY-AngII, when we conducted the NanoBRET assay using cells transfected with only Nluc-β_2_AR and treated with BODIPY-AngII in the presence or absence of olmesartan (Figure 2j), no statistically significant specific binding of BODIPY-AngII was observed (Figure 2k,l).

Although cells expressing Nluc-AT_1_ (Figure 2b) and Nluc-β_2_AR + AT_1_ (Figure 2h) displayed saturable binding of BODIPY-AngII that could be displaced by olmesartan, there still appeared to be a saturable component to the BODIPY-AngII binding that was not displaced by olmesartan. This saturable binding is likely due to the presence of a non AT_1_ receptor AngII-binding site, and as to our knowledge the AT_2_ receptor is not endogenously expressed in HEK293 cells, this binding site may be a non-AT_1_, non-AT_2_ receptor AngII-binding site that has previously been detected in HEK293 cells [36]. Wangler et al. [36] observed saturable binding of ^125^I-SI-AngII in HEK293 cells, which could not be displaced by either AT_1_ or AT_2_ receptor blockade, and they identified this non-AT_1_, non-AT_2_ receptor AngII-binding site as the membrane-bound variant of metalloendopeptidase neurolysin. It is possible that in our assays this protein is in sufficiently close proximity to Nluc-AT_1_ (Figure 2b) or Nluc-β_2_AR + AT_1_ (Figure 2h) to produce a NanoBRET signal and saturable BODIPY-AngII binding not able to be displaced by olmesartan.

**Figure 3:**
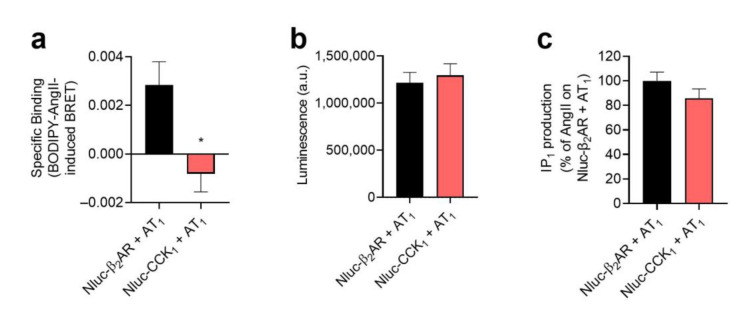
Lack of Receptor-Heteromer Investigation Technology (Receptor-HIT) signal between AT_1_ and CCK_1_. Cells were transfected with Nluc-β_2_AR + AT_1_ or Nluc-CCK_1_ + AT_1_. In the NanoBRET ligand binding assay (**a**), cells were treated with 1 μM BODIPY-AngII in the presence or absence of 1 μM olmesartan to enable calculation of BODIPY-AngII specific binding. In the luminescence assay (**b**), raw luminescence values were obtained from untreated aliquots of cells. In the IP_1_ assay (**c**), cells were treated with 1 μM AngII. Significant differences (*) were observed in (**a**) but not (**b**) or (**c**), using unpaired *t*-tests. Data are displayed as mean ± SEM of three independent experiments.

To confirm the specificity of the NanoBRET signal, we investigated binding of BODIPY-AngII in cells expressing a Nluc-tagged GPCR not known to heteromerize with the AT_1_ receptor, the cholecystokinin CCK_1_ receptor. In contrast to the specific binding we observed in Nluc-β_2_AR + AT_1_ cells, we did not observe specific binding of BODIPY-AngII in cells expressing Nluc-CCK_1_ + AT_1_ (Figure 3a). This lack of specific binding was unlikely to be due to decreased expression of Nluc-CCK_1_ compared to Nluc-β_2_AR, as there was no significant difference observed between the luminescence produced by both sets of cells (Figure 3b). Additionally, the lack of specific binding in Nluc-CCK_1_ + AT_1_ cells was unlikely to be due to decreased expression of the AT_1_ receptor, as there was no significant difference in the level of IP_1_ produced between the two sets of cells upon stimulation with AngII (Figure 3c). Thus, this indicates that the specific binding observed in Nluc-β_2_AR + AT_1_ cells is due to binding of BODIPY-AngII to AT_1_ receptors heteromerized with Nluc-β_2_AR, and not due to overcrowding of receptors at the plasma membrane.

**Figure 4:**
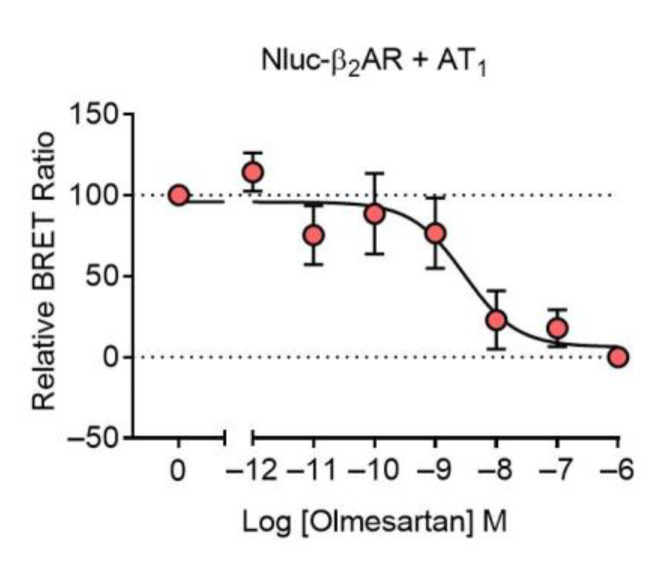
Competition binding of BODIPY-AngII to the AT_1_-β_2_AR heteromer. Cells expressing Nluc-β_2_AR + AT_1_ were treated with 500 nM BODIPY-AngII in the presence of increasing concentrations of olmesartan. Data are normalized (100% being vehicle-treated and 0% being 1 μM olmesartan-treated) and displayed as relative BRET ratio, mean ± SEM of four independent experiments.

We next wanted to see if we could also detect binding at AT_1_-β_2_AR heteromers using a BRET competition binding assay. In cells expressing Nluc-β_2_AR + AT_1_ there was clear binding of BODIPY-AngII, which could be displaced by increasing concentrations of olmesartan (Figure 4). This confirmed that the NanoBRET ligand binding assay could be run successfully as a competition assay as well as a saturation assay.

**Figure 5:**
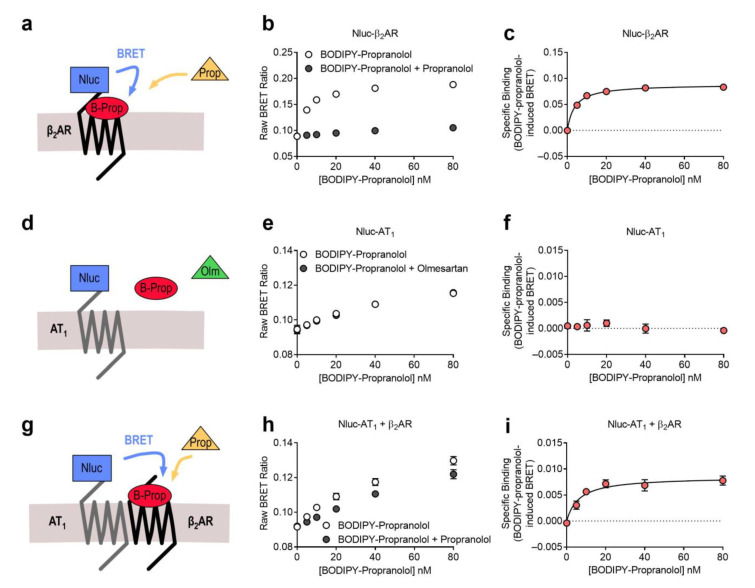
Saturation binding of BODIPY-propranolol to the β_2_AR and the AT_1_-β_2_AR heteromer. Cells expressing Nluc-β_2_AR (**a**–**c**) or Nluc-AT_1_ (**d**–**f**) or Nluc-AT_1_ + β_2_AR (**g**–**i**) were treated with BODIPY-propranolol (B-Prop) in the presence or absence of 10 μM propranolol (Prop; **a**–**c**,**g**–**i**) or the presence or absence of 1 μM olmesartan (Olm; **d**–**f**) to generate BODIPY-propranolol total and non-specific binding data (**b**,**e**,**h**) and specific binding curves (where possible; **c**,**f**,**i**). Data are displayed as the raw BRET ratio (**b**,**e**,**h**) or specific binding (**c**,**f**,**i**), mean ± SEM of three (**e**,**f**), four (**b**,**c**) or eight (**h**,**i**) independent experiments.

We then wanted to investigate the results of the AT_1_-β_2_AR heteromer ligand binding assay when it was conducted in the reverse configuration. For these assays we used a BODIPY-630/650 tagged propranolol ligand (BODIPY-propranolol). When we investigated binding to Nluc-β_2_AR (Figure 5a), we observed saturable binding of BODIPY-propranolol that could be completely displaced by unlabeled propranolol (Figure 5b), enabling generation of a BODIPY-propranolol specific binding curve (Figure 5c). When we conducted the assay using cells expressing Nluc-AT_1_ (Figure 5d), we did not observe specific binding of BODIPY-propranolol to the AT_1_ receptor that was sensitive to displacement by olmesartan (Figure 5e,f). Next, we conducted the assay using the Receptor-HIT configuration, with cells expressing Nluc-AT_1_ + β_2_AR (Figure 5g). Here we observed displacement of BODIPY-propranolol binding in the presence of unlabeled propranolol (Figure 5h) resulting in generation of a BODIPY-propranolol specific binding curve (Figure 5i), confirming the close proximity of the two receptors in our system. Similar to what we observed in the previous configuration (Nluc-β_2_AR + AT_1_), the BODIPY-propranolol-induced BRET signal between BODIPY-propranolol and β_2_AR was approximately ten times stronger than between BODIPY-propranolol and AT_1_-β_2_AR heteromers, again likely due to the increased distance when BODIPY-propranolol binds to β_2_ARs that are heteromerized with Nluc-AT_1_. Interestingly, and similar to what was observed in the previous configuration, when we compare the affinity of BODIPY-AngII binding in the two systems, we see a small but significant decrease in the affinity of binding to AT_1_-β_2_AR heteromers when compared to binding to β_2_ARs (mean *K*_d_ ± SEM = 8.8 ± 4.0 nM n = 8 vs. 3.9 ± 0.5 nM *n* = 4, respectively; *p* < 0.05 for unpaired *t*-test).

**Figure 6:**
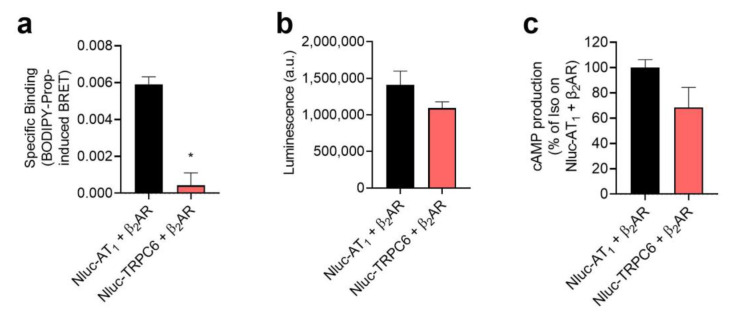
Lack of Receptor-Heteromer Investigation Technology (Receptor-HIT) signal with β_2_AR and TRPC6. Cells were transfected with Nluc-AT_1_ + β_2_AR or Nluc-TRPC6 + β_2_AR. In the BRET ligand binding assay (**a**), cells were treated with 80 nM BODIPY-propranolol in the presence or absence of 10 μM propranolol to enable calculation of BODIPY-propranolol specific binding. In the luminescence assay (**b**), raw luminescence values were obtained from untreated aliquots of cells. In the cAMP assay (**c**), cells were treated with 10 μM isoprenaline (Iso). Significant differences (*) were observed in (**a**) but not (**b**,**c**), using unpaired *t*-tests. Data are displayed as mean ± SEM of three independent experiments.

As with the previous configuration, we needed to confirm that the Receptor-HIT signal we observed was not due to overcrowding of receptors at the cell surface. Again we used a control receptor, this time the TRPC6 transient receptor potential channel 6 (TRPC6). Here we saw that there was no specific binding of BODIPY-propranolol in cells expressing Nluc-TRPC6 + β_2_AR (Figure 6a), unlike cells expressing Nluc-AT_1_ + β_2_AR. This lack of signal was unlikely to be due to a reduced expression of Nluc-TRPC6 relative to Nluc-AT_1_, as there was no significant difference in the levels of luminescence produced (Figure 6b). Nor was the lack of signal likely to be due to reduced expression of β_2_AR, as there was no significant difference in the level of isoprenaline-induced cAMP production by both sets of cells (Figure 6c). Thus, this indicates that the specific binding observed in Nluc-AT_1_ + β_2_AR cells is due to binding of BODIPY-propranolol to β_2_ARs that form heteromers with Nluc-AT_1_, and not due to overcrowding of receptors at the plasma membrane.

**Figure 7:**
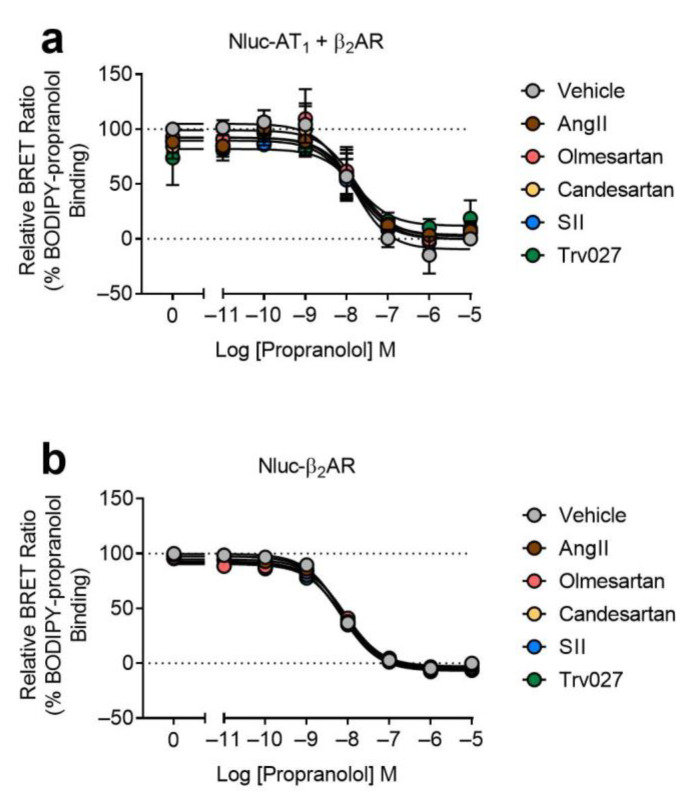
Competition binding of BODIPY-propranolol to β_2_AR and the AT_1_-β_2_AR heteromer. Cells expressing Nluc-AT_1_ + β_2_AR (**a**) or Nluc-β_2_AR (**b**) were treated with 50 nM BODIPY-propranolol in the presence of various AT_1_ ligands (1 μM) and increasing concentrations of propranolol. Data are normalized to vehicle treatment for that transfection (0% being 10 μM propranolol-treated and 100% being vehicle only treated) and displayed as relative BRET ratio, mean ± SEM of four independent experiments.

Finally, we wanted to investigate if there were any changes to the binding of BODIPY-propranolol when the AT_1_ protomer of the AT_1_-β_2_AR heteromer was bound by a selective ligand. To do this, we investigated the effects of various AT_1_ receptor ligands on propranolol-induced displacement of BODIPY-propranolol binding in a competition assay (Figure 7a). This showed that there was no significant change in the pIC_50_ values of displacement of BODIPY-propranolol bound to AT_1_-β_2_AR heteromers (Figure 7a and Table 1) or β_2_AR protomers (Figure 7b and Table 1).

### 2.2. AT_1_-AT_2_ Heteromer

**Figure 8:**
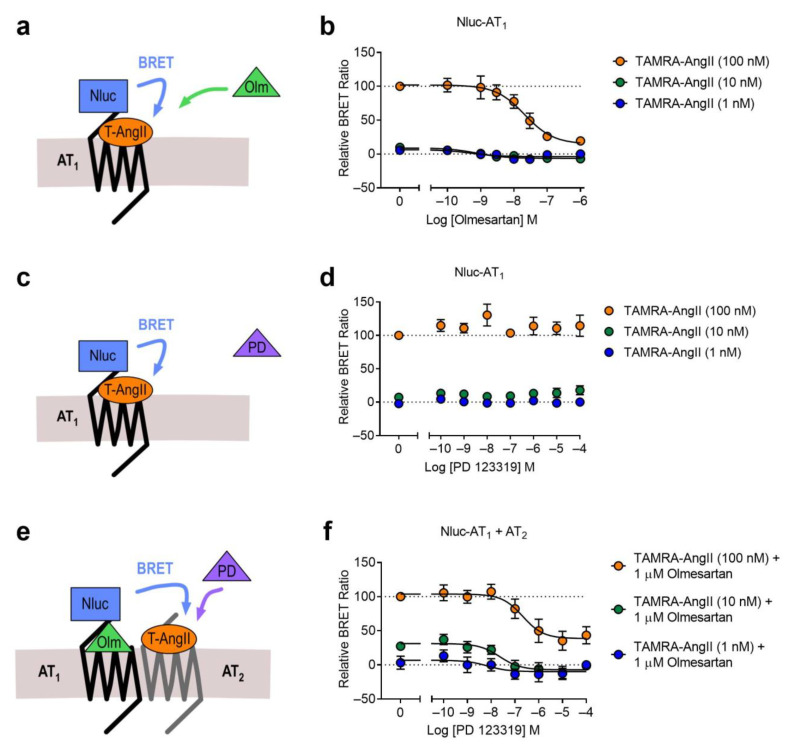
Competition binding of TAMRA-AngII to the AT_1_ receptor and the AT_1_-AT_2_ heteromer. Cells expressing Nluc-AT_1_ and treated with various concentrations of TAMRA-AngII (T-AngII) as stated, with increasing concentrations of olmesartan (Olm) (**a**,**b**). Cells expressing Nluc-AT_1_ and treated with various concentrations of TAMRA-AngII as stated, with increasing concentrations of PD 123319 (PD) (**c**,**d**). Cells expressing Nluc-AT_1_ and untagged AT_2_ and treated with 1 μM olmesartan, various concentrations of TAMRA-AngII as stated, and increasing concentrations of PD 123319 (**e**,**f**). Data are normalized (100% being 100 nM TAMRA-AngII treated with no competitor present and 0% being 1 nM TAMRA-AngII treated at maximum competitor concentration) and displayed as relative BRET ratio, mean ± SEM of three independent experiments.

In addition to the AT_1_-β_2_AR heteromer, we also used the NanoBRET ligand binding assay to investigate heteromer formation between the AT_1_ receptor and the AT_2_ receptor, both of which bind AngII. This heteromer has been well characterized in numerous systems, and has been shown to cause alterations to receptor signaling, β-arrestin recruitment and trafficking [4,26,27,28,29,30,31,32,33,34,35]. We investigated this heteromer by first looking at binding of various concentrations of a TAMRA-labeled AngII (TAMRA-AngII) to Nluc-AT_1_ in a competition binding assay with olmesartan (Figure 8a). Here we observed displacement of TAMRA-AngII bound to Nluc-AT_1_ with olmesartan (Figure 8b). We then conducted a similar assay using the AT_2_ selective antagonist PD 123319 (Figure 8c), and this time we were unable to see any displacement of bound TAMRA-AngII (Figure 8d). Finally, we conducted the assay in the Receptor-HIT configuration to look for proximity between the two receptors, by coexpressing Nluc-AT_1_ with the untagged AT_2_ receptor (Figure 8e). However, due to the non-selectivity of the TAMRA-AngII ligand we needed to block the Nluc-tagged AT_1_ receptors to prevent their binding of TAMRA-AngII. To do this we treated the cells with 1 μM olmesartan, the concentration at which we saw complete displacement of TAMRA-AngII binding previously (Figure 8b). In addition to olmesartan, cells were treated with TAMRA-AngII and increasing concentrations of PD 123319, to see if we were able to observe binding of TAMRA-AngII that could be displaced by PD 123319. This was indeed the case (Figure 8f), thus confirming that TAMRA-AngII binding was occurring specifically at AT_2_ receptors, and not AT_1_ receptors. This indicated that Nluc-AT_1_ was proximal to TAMRA-AngII-bound AT_2_ receptors, again confirming that the Receptor-HIT assay could be used successfully to monitor receptor heteromers at their extracellular surface.

**Figure 9:**
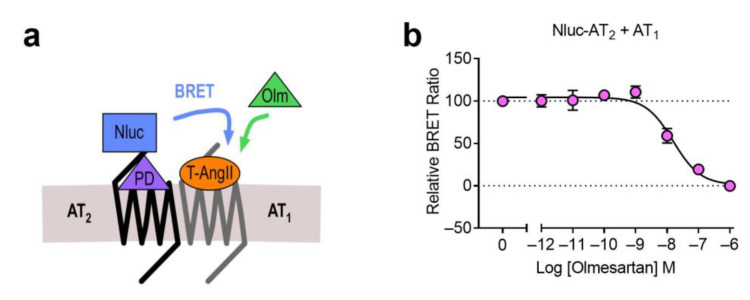
Competition binding of TAMRA-AngII to the AT_1_-AT_2_ heteromer. Cells expressing Nluc-AT_2_ and untagged AT_1_ (**a**) were treated with 100 µM PD 123319 (PD), 1 µM TAMRA-AngII (T-AngII) and increasing concentrations of olmesartan (Olm) (**b**). Data are normalized (100% being vehicle treated and 0% being 1 µM olmesartan treated) and displayed as relative BRET ratio, mean ± SEM of three independent experiments.

Finally, we repeated the assay in the reverse configuration. Here, Nluc-AT_2_ was coexpressed with the untagged AT_1_ receptor, and we used 100 μM PD 123319 to block Nluc-AT_2_ (Figure 9a). Treatment with TAMRA-AngII, and increasing concentrations of olmesartan showed displacement of TAMRA-AngII bound specifically to AT_1_ receptors (Figure 9b). This indicated the close proximity of AT_1_ receptors to Nluc-AT_2_, and confirmed that the assay could be used successfully in both configurations.

## 3. Discussion

In this study we have successfully adapted the NanoBRET ligand binding assay to investigate heteromers using the Receptor-HIT configuration. This demonstrates broadening of the applicability of Receptor-HIT using BRET, so that it can now be used to provide information about heteromer ligand binding, as well as heteromer interactions with intracellular proteins.

Traditionally, evidence for GPCR heteromerization from ligand binding studies has come from radioligand binding assays that reveal ligand cooperativity upon coexpression of the two receptors of interest [37]. Indeed, there are numerous examples of studies providing evidence for GPCR oligomers that display either positive [38,39,40] or negative [41,42,43,44] ligand cooperativity. While this approach enables elucidation of functional consequences of oligomerization, careful interpretation of the data is required to ensure the observed results are actually a consequence of heteromer-mediated cooperativity. For example, as GPCRs can have different affinities for ligands when they are bound to G proteins, dual receptor activation could limit the supply of G proteins, leading to altered ligand affinities and thus false ligand cooperativities [37]. Furthermore, assessment of ligand cooperativity does not provide direct evidence for proximity between receptors, and nor will it enable identification of ligand interactions with neutral cooperativity. The Receptor-HIT ligand binding assay overcomes these limitations as it is able to detect heteromers based on receptor proximity, not solely on ligand cooperativity. Additionally, using a proximity assay such as BRET with fluorescent ligands overcomes all of the issues associated with the use of radiolabeled ligands, which are costly, time consuming to use, require numerous measures to monitor and minimize exposure to radioactivity when carrying out the assays, and generate radioactive waste that requires appropriate disposal.

Previously, the Receptor-HIT approach using time resolved fluorescence resonance energy transfer (TR-FRET) has been utilized to investigate ligand binding to the dopamine D_1_-D_3_ heteromer. Hounsou et al. [45] coexpressed SNAP-tag-labeled D_1_ receptors (SNAP-D_1_) with D_3_ receptors, and treated with a fluorescent D_3_ receptor-selective ligand. This approach worked well and they were able to measure TR-FRET between SNAP-D_1_ and the fluorescent ligand bound to the D_3_ receptor. Furthermore, these investigators were able to confirm that the fluorescent ligand was binding specifically to the D_3_ receptor through displacement with D_3_ selective ligands. While both the TR-FRET and our BRET approach were successful, our technique has one significant advantage over TR-FRET. Although both assays require the addition of substrates for the energy donor (furimazine for Nluc and SNAP-Lumi4-Tb for TR-FRET [46]), the TR-FRET method requires removal of the SNAP-tag substrate and several wash steps. In contrast, the BRET assay can be performed in a completely homogenous manner, reducing time and potential introduction of error that can occur through multiple wash steps.

Extending its application beyond in vitro systems, NanoBRET has now also been used to visualize ligand binding to a GPCR in vivo. MDA-MB-231 triple-negative human breast cancer cells stably expressing Nluc-β_2_AR were injected into a mammary fat pad of a mouse [47]. Once the tumor size reached over 200 mm^3^, BRET ratios indicating ligand binding were determined following administration of BODIPY-propranolol. Future studies could potentially use the Receptor-HIT configuration with such an approach to investigate receptor heteromers in vivo.

More recently, NanoBRET ligand binding has been demonstrated at a GPCR under endogenous promotion facilitated by CRISPR/Cas9 genome editing [21]. Again, such an approach could enable the Receptor-HIT ligand binding assay to be utilized for GPCRs under endogenous promotion, as has already been done for Receptor-HIT using proximity to the intracellular β-arrestin molecule, albeit when the arrestin and the second receptor were exogenously expressed [10].

Proximity ligation assays are an alternative to BRET, and have been used to visualize protein–protein interactions in primary tissue, including investigating heteromerization of GPCRs [48,49,50]. However, these assays require well-validated antibodies, which are not always available for GPCRs in particular [17].

The AT_1_-β_2_AR heteromer ligand binding assay revealed that there was a far greater level of BRET produced when the fluorescent ligand was bound directly to the Nluc-tagged receptor than when the fluorescent ligand was bound to the untagged receptor complexed with the Nluc-tagged receptor. The major cause of this increase is likely to be due to the difference in distance between the BRET donor and acceptor. BRET efficiency is inversely proportional to the distance between the donor and acceptor molecules to the sixth power, resulting in energy transfer that occurs over distances of less than about 10 nm [51,52]. As GPCRs are estimated to be ~4 nm in diameter [53], this highlights the sensitivity of the BRET assay at detecting small changes in proximity between donor and acceptor molecules. Another potential contributing factor to the increased level of BRET in the monomer/homomer assay is that the proportion of heteromers in the system may be lower than monomers/homomers. Although most studies suggest that GPCRs display a similar propensity to form homomers or heteromers [54], there are examples of receptors that reportedly have greater [55,56] or lesser [57,58] proclivity to form homomers relative to heteromers.

The results of this study showed a decrease in affinity of both fluorescent ligands when bound to receptors within the AT_1_-β_2_AR heteromer when compared to either receptor alone. This suggests that the unbound receptor within the heteromer may be allosterically modulating the bound receptor, causing a reduction in the affinity of the fluorescent ligand. This is a novel finding for the AT_1_-β_2_AR heteromer, as ligand binding has not been previously investigated for this complex.

As well as the AT_1_-β_2_AR heteromer, we were also able to successfully use the heteromer ligand binding assay to investigate the AT_1_-AT_2_ heteromer. As the TAMRA-AngII ligand could bind to both receptors, this assay required a more complicated set up, with the Nluc-labeled receptor blocked from TAMRA-AngII binding with the use of a selective antagonist. Nevertheless, we were still able to clearly see binding of TAMRA-AngII to the unlabeled receptor, indicating its heteromerization with the Nluc-tagged receptor. It should be noted however, that ligand binding to one receptor may allosterically alter binding affinity to the second receptor within the heteromer, and this should therefore be taken into consideration when calculating ligand affinities with this assay set up.

In conclusion, we have demonstrated that the Receptor-HIT assay can be used with the NanoBRET ligand binding assay to detect receptor heteromerization. We have successfully monitored the proximity between receptors within the AT_1_-β_2_AR heteromer and the AT_1_-AT_2_ heteromer, the latter being a complex that can be interrogated with a non-selective fluorescent ligand with the additional use of unlabeled selective antagonists. We have also demonstrated that this real-time, live cell, non-radioactive assay is sensitive enough to perform completely homogenously, further increasing its exciting potential for drug discovery and profiling.

## 4. Materials and Methods

### 4.1. cDNA Constructs

Nluc-β_2_AR [18], Nluc-AT_1_ [18] and Nluc-CCK_1_ were from Promega (Madison, WI, USA). Nluc-TRPC6 was generated by GeneArt (Thermo Fisher Scientific, Regensburg, Bavaria, Germany). Nluc-AT_2_ was generated by inserting the AT_2_ coding region (obtained from the Missouri S&T cDNA Resource Center (Rolla, MO, USA) into a pcDNA3.Nluc vector generated in house (containing the IL6 signal peptide upstream to Nluc). The β_2_AR construct was from the Missouri S&T cDNA Resource Center. The human AT_1_ and AT_2_ receptor constructs used were generated by inserting the AT_1_ or AT_2_ coding region (obtained from the Missouri S&T cDNA Resource Center) after the mGluR5 signal peptide and FLAG-tag coding region in a pcDNA3 construct made in house.

### 4.2. Ligands, and Generation of BODIPY-AngII

TAMRA-AngII was from AnaSpec (Fremont, CA, USA), BODIPY-propranolol (CA200689) was from Hello Bio (Bristol, UK), olmesartan medoxomil was from Zhou Fang Pharm Chemical (Shanghai, China) and Sigma Aldrich (Castle Hill, NSW, Australia). AngII, candesartan cilexetil and PD 123319 were from Sigma Aldrich.

BODIPY-AngII [19], SII and Trv0027 were made by solid phase peptide synthesis. The amino acid sequence for each peptide was assembled on 2-chlorotrityl resin (0.8 mmol/g loading) using standard Fmoc chemistry with HBTU/DIEA activation. Briefly, 2-chlorotrityl resin was swelled in dichloromethane (DCM) for 1 h, then 2 equiv of Fmoc-protected C-terminal amino acid with 8 equiv of DIEA were added to the resin and allowed to react for 30 min. Unreacted sites were blocked with DCM/MeOH/DIEA (17:2:1). The N-terminal Fmoc protection group was removed with 20% piperidine in dimethylformamide (2 × 5 min). All remaining couplings were performed on a CS Bio CS336X automated synthesizer with 4 equiv of Fmoc-amino acid, 8 equiv of HBTU and 8 equiv of DIEA. The peptide was cleaved from the resin with TFA/TIPS/Milli Q (95:2.5:2.5) and lyophilized. The crude peptide was dissolved in 10% acetonitrile and purified by RP-HPLC on a Prominence HPLC system (Shimadzu) using a semipreparative Grace Vydac C18 column (250 mm × 10 mm, 10 μm) at a 1% gradient and a flow rate of 3 mL/min. Electrospray mass spectrometry (AB Sciex) was used to confirm the molecular weight. For N-terminal labeling the pure lyophilized AngII was dissolved in DMF at 1 mM concentration with 100 mM trimethylamine and 1 mM of BODIPY^TM^ 630/650-x NHS ester (Thermo Fisher Scientific, Brisbane, Australia), and stirred for 24 h, protected from light. The labeling reaction was monitored with analytical RP-HPLC and electrospray mass spectrometry.

### 4.3. Cell Culture and Transfection

HEK293FT cells were maintained at 37 °C, 5% CO_2_ in complete medium (Dulbecco’s modified Eagle’s medium (DMEM) containing 0.3 mg/mL glutamine, 100 IU/mL penicillin and 100 μg/mL streptomycin) supplemented with 10% fetal calf serum (FCS) (GIBCO BRL, Carlsbad, CA, USA). Transient transfections were carried out directly in a 96-well plate using FuGENE 6 (Promega) according to the manufacturer’s instructions. The cDNA transfection mix was added to 100,000 cells/well in DMEM supplemented with 10% FCS. Cells were then incubated at 37 °C, 5% CO_2_, and assays were carried out 48 h post transfection.

### 4.4. NanoBRET Saturation Ligand Binding Assays

Saturation ligand binding assays (Figure 2, Figure 3, Figure 5 and Figure 6) were conducted by firstly removing the media and replacing with vehicle (HBSS) or competitor ligand diluted in HBSS. This was incubated for 20 min at 37 °C, 5% CO_2_ and then the fluorescent ligands diluted in HBSS were added and incubated for a further 40 min. Furimazine (10 µM) was then added and luminescence was measured immediately at 37 °C using the LUMIstar plate reader (BMG Labtech, Mornington, VIC, Australia) with 450 nm (80-nm bandpass) and >610 nm (longpass). The raw BRET ratio was calculated by dividing the long wavelength emission by the short wavelength emission. Non-specific binding was determined in the presence of 1 µM olmesartan or 10 µM propranolol as indicated. Specific binding was then calculated by subtracting the raw BRET ratio of the competitor-treated sample (non-specific binding) from the raw BRET ratio of the vehicle treated sample (total binding). All individual experiments were conducted with duplicate wells.

### 4.5. NanoBRET Competition Ligand Binding Assays

Competition ligand binding assays (Figure 4, Figure 7, Figure 8 and Figure 9) were conducted by firstly removing the media and replacing with vehicle (HBSS) or competitor ligand(s) diluted in HBSS. This was incubated for 20 or 30 min at 37 °C, 5% CO_2_ and then the fluorescent ligands diluted in HBSS were added and incubated for a further 30 or 40 min. Furimazine (10 µM) was then added and luminescence was measured immediately at 37 °C using either the PHERAstar *FS* plate reader (BMG Labtech) with 460 nm (80-nm bandpass) and >610 nm (longpass) or the LUMIstar plate reader (BMG Labtech) with 450 nm (80-nm bandpass) and >610 nm (longpass). The relative BRET ratio was calculated by normalizing the data, as described in the figure legends. All individual experiments were conducted with duplicate wells.

### 4.6. Inositol Monophosphate (IP_1_) Accumulation Assays

Measurement of IP_1_ accumulation was performed using the IP-One Tb kit (Cisbio Bioassays (PerkinElmer), Codolet, Occitania, France) according to the manufacturer’s instructions. Cells were treated for 30 min at 37 °C with AngII or vehicle. The cells were then lysed by adding the supplied assay reagents, and the assay was incubated for 1 h at room temperature. Fluorescence was measured at 620 nm and 665 nm, 50 µs after excitation at 340 nm using the EnVision 2102 multilabel plate reader (PerkinElmer, Glen Waverley, VIC, Australia).

### 4.7. cAMP Accumulation Assays

Measurement of cAMP accumulation was performed using the cAMP dynamic 2 assay kit (Cisbio Bioassays) according to the manufacturer’s instructions. Cells were treated for 30 min at 37 °C with isoprenaline or vehicle. The cells were then lysed by adding the supplied assay reagents, and the assay was incubated for 1 h at room temperature. Fluorescence was measured at 620 nm and 665 nm, 50 μs after excitation at 340 nm using the EnVision 2102 multilabel plate reader (PerkinElmer).

### 4.8. Data Presentation and Statistical Analysis

All data were presented and analyzed using GraphPad Prism 8.0. Saturation binding data were fitted with one-site nonlinear regression for specific binding. Competition binding data were fitted using logarithmic nonlinear regression (three parameter). Statistical analysis was performed as described in the relevant figure/table legends or in the text, with *p* < 0.05 considered significant.

## Figures and Tables

**Table 1:** pIC_50_ values from competition curves in Figure 7. No significant differences were observed within each transfection using one-way ANOVA.

	Vehicle	AngII	Olmesartan	Candesartan	SII	Trv027
Nluc-AT_1_ + β_2_AR	7.96 ± 0.18	7.94 ± 0.31	7.91 ± 0.26	7.92 ± 0.32	7.98 ± 0.31	7.88 ± 0.35
Nluc-β_2_AR	8.17 ± 0.02	8.14 ± 0.02	8.06 ± 0.02	8.10 ± 0.04	8.16 ± 0.11	8.12 ± 0.05

## Data Availability

The data that support the findings of this study are available from the corresponding author upon reasonable request.
